# Ferroptosis of Endothelial Cells in Vascular Diseases

**DOI:** 10.3390/nu14214506

**Published:** 2022-10-26

**Authors:** Hanxu Zhang, Shuang Zhou, Minxue Sun, Manqi Hua, Zhiyan Liu, Guangyan Mu, Zhe Wang, Qian Xiang, Yimin Cui

**Affiliations:** 1 Department of Pharmacy, Peking University First Hospital, Beijing 100034, China; 2 School of Pharmaceutical Sciences, Peking University Health Science Center, Beijing 100191, China; 3 School of Basic Medicine and Clinical Pharmacy, China Pharmaceutical University, Nanjing 211198, China; 4 Institute of Clinical Pharmacology, Peking University, Beijing 100191, China

**Keywords:** ferroptosis, endothelial cell, vascular disease

## Abstract

Endothelial cells (ECs) line the inner surface of blood vessels and play a substantial role in vascular biology. Endothelial dysfunction (ED) is strongly correlated with the initiation and progression of many vascular diseases. Regulated cell death, such as ferroptosis, is one of the multiple mechanisms that lead to ED. Ferroptosis is an iron-dependent programmed cell death associated with various vascular diseases, such as cardiovascular, cerebrovascular, and pulmonary vascular diseases. This review summarized ferroptosis of ECs in vascular diseases and discussed potential therapeutic strategies for treating ferroptosis of ECs. In addition to lipid peroxidation inhibitors and iron chelators, a growing body of evidence showed that clinical drugs, natural products, and intervention of noncoding RNAs may also inhibit ferroptosis of ECs.

## 1. Introduction

Ferroptosis is an iron-dependent programmed cell death primarily characterized by reduced cystine uptake into cells, followed by glutathione (GSH) depletion, iron-dependent lipid peroxidation, and release of damage-associated molecular patterns [1,2]. Cell volume shrinkage and mitochondrial membrane thickening are the predominant morphologic hallmarks of ferroptosis [3]. Dixon et al. defined ferroptosis for the first time in 2012 [1]. It has since become a hot area of research. Accumulating evidence indicates that ferroptosis is linked to various pathological states, such as tumors, cardiovascular diseases, cerebrovascular disease, acute lung injury (ALI), and neurodegenerative disorders.

Endothelial cells (ECs) have direct contact with chemicals or particles in the circulatory system. The vascular endothelium is essential for maintaining multiorgan health and homeostasis, including the dynamic maintenance of vasodilation and vasoconstriction, angiogenesis and anti-angiogenesis, antithrombosis and pro-thrombosis, antiinflammation and proinflammation, and antioxidation and pro-oxidation [4,5,6,7,8]. Endothelial dysfunction (ED) is highly correlated with diverse human pan-vascular diseases, including atherosclerosis (AS), hypertension, diabetes, and ALI [9,10], and contributes to the development of neurodegenerative diseases such as Alzheimer’s disease [11]. Due to its essential role in many diseases, the endothelium is a therapeutic target for the prevention and treatment of the above pan-vascular diseases.

Regulated cell death (RCD), such as ferroptosis, occurring in ECs is one of the multiple mechanisms that lead to ED. Evidence suggests that inhibiting ferroptosis can attenuate ED [12]. Therefore, ferroptosis may be a promising target to protect the endothelium. This review aimed to highlight the effects of ferroptosis in ECs, summarize new treatments based on ferroptosis of ECs, and identify research trends and hotspots in this field.

## 2. Mechanism of Ferroptosis

RCD is essential in all aspects of life and contributes to various human diseases under different stresses [13,14]. A genetically encoded apparatus initiates RCD, which can be altered by pharmacologic or genetic intervention. Thus, there is a growing interest in modulating RCD to treat diseases. Due to differences in morphology, biochemistry, and genetic levels, RCD can be classified into various subtypes (necroptosis, apoptosis, autophagy, pyroptosis, ferroptosis, etc.) [14,15,16,17,18,19], as shown in Table 1.

Ferroptosis is primarily mediated by iron metabolism and lipid peroxidation [20]. It occurs as a result of iron-catalyzed lipid peroxidation, which can be initiated by both enzymatic and nonenzymatic mechanisms, such as the Fenton reaction. Two enzymes, lysophosphatidylcholine acyltransferase 3 (LPCAT3) and acyl-CoA synthetase long-chain family member 4 (ACSL4), contribute to the biosynthesis and remodeling of polyunsaturated fatty acids (PUFAs)-phosphatidylethanolamines in cellular membranes [2]. PUFAs produce lipid hydroperoxides, as well as toxic lipid free radicals, which transfer protons near PUFAs and trigger a new round of lipid oxidation reactions [21].

Ferroptosis can be caused by iron overload triggered by an increase in iron uptake via transferrin (TF) receptor 1 (TFR1) and a reduction in iron export via ferroportin 1. Ferrous ions (Fe^2+^) mediate the Fenton reaction, which can increase reactive oxygen species (ROS) and result in ferroptosis [21,22,23]. In addition, some Fe^2+^ are stored in ferritin. Ferritinophagy, which converts ferritin into intracellular iron through nuclear receptor coactivator 4 (NCOA4), also results in ferroptosis [24,25,26,27].

**Figure 1:**
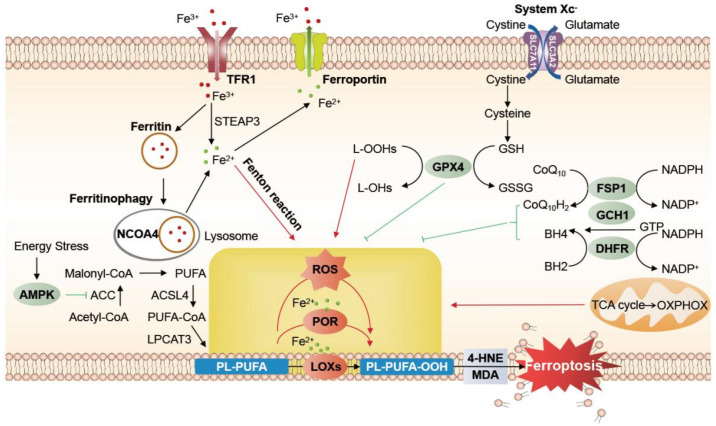
Key mechanisms of ferroptosis. The regulatory pathways of ferroptosis are mainly divided into three metabolisms (iron, lipid, and amino acid). By binding TFR1, cells take iron into endosomes as Fe^3+^. Six-transmembrane epithelial antigen of prostate 3 (STEAP3) helps reduce Fe^3+^ to Fe^2+^. Most Fe^2+^ can be stored in ferritin, utilized for enzyme synthesis, or exported by ferroportin (oxidized to Fe^3+^). The rest of Fe^2+^ may mediate Fenton reaction producing ROS. The accumulation of ROS will induce ferroptosis because it oxidizes PL-PUFA-OH into PL-PUFA-OOH, which can decompose to 4-hydroxy-2-nonenals (4-HNEs) or malondialdehydes (MDAs) that cause cellular damage. Another important cause of ferroptosis is reduced antioxidant capacity. The system Xc^−^ transporter, consisting of solute carrier family 7 member 11 (SLC7A11) and solute carrier family 3 member 2 (SLC3A2), releases glutamate in exchange for cystine, which can be further transformed into cysteine, a crucial component of GSH. With the help of GPX4, GSH can deoxidize PL-PUFA-OOH back into PL-PUFA-OH. GPX4 can inhibit ferroptosis by reducing ROS production and protecting cells from lipid hydroperoxides. In addition, the FSP1-CoQ_10_-NAD(P)H pathway, GCH1-BH4-DHFR pathway, mitochondria, and energy stress are involved in the progress of ferroptosis as well. GSSG: glutathione disulfide; L: lipid; -OHs: alcohols; -OOHs: hydroperoxides; CoA: coenzyme A; ACC: acetyl coenzyme A carboxylase; PL: phospholipid; POR: cytochrome P450 reductase; LOXs: lipoxygenases; TAC: tricarboxylic acid; OXPHOX: oxidative phosphorylation; CoQ_10_H_2_: the reduced form of coenzyme Q_10_, ubiquinol; NADH: nicotinamide adenine dinucleotide; NADPH: nicotinamide adenine dinucleotide phosphate; BH2: dihydrobiopterin.

System Xc^−^ mediates the exchange of cystine and glutamate in a 1:1 ratio. Erastin induces ferroptosis by inhibiting system Xc^−^. Glutathione peroxidase 4 (GPX4) is a selenoenzyme that lowers ROS via GSH and protects lipid peroxidation [28]. It is crucial in the regulation of ferroptosis and atherosclerosis [29]. Ras-selective lethal small molecule 3 (RSL3) impairs antioxidant capacity by inhibiting GPX4, ultimately causing ferroptosis. Several studies have reported that p53 can decrease solute carrier family 7 member 11 (SLC7A11) expression, inhibit GPX4 activity, and lead to ferroptosis [30]. SLC7A11 and GPX4 levels are increased by nuclear factor erythroid 2-related factor 2 (NRF2), and therefore reduces ROS. NRF2 overexpression inhibits ferroptosis, whereas Kelch-like ECH-associated protein 1 (Keap 1), which binds to NRF2 and negatively regulates it, can reverse this process [31,32]. Activating the KEAP1/NRF2 pathway can cause ferroptosis by upregulating heme oxygenase 1 (HMOX1) [33]. Moreover, the ferroptosis suppressor protein 1 (FSP1)-coenzyme Q_10_ (CoQ_10_)-nicotinamide adenine dinucleotide (phosphate) (NAD(P)H) pathway [34,35], guanosine 5′-triphosphate cyclohydrolase 1 (GCH1)-tetrahydrobiopterin (BH4)-dihydrofolate reductase (DHFR) pathway [36,37], adenosine monophosphate-activated protein kinase (AMPK) [38,39], and mitochondria [40] also play vital roles in eliminating excess lipid peroxides and regulating ferroptosis. Key molecular mechanisms of ferroptosis are summarized in Figure 1.

## 3. Endothelial Dysfunction

ECs perform a variety of key homeostatic functions [6]. However, ED is characterized by a change from executing physiological functions of the vascular endothelium to inflammation, hyperpermeability, leukocyte adhesion, endothelial nitric oxide synthase uncoupling, altered EC metabolism, oxidative stress, vasoconstriction, injury, cell death, senescence, and endothelial-to-mesenchymal transition [9]. In a wide range of circumstances, ED, which is usually caused by EC injury or death, is clearly associated with poor prognosis. Oxidative stress is one of the most important underlying mechanisms for ED development [41]. In cardiovascular diseases, multiple risk factors that upregulate intracellular oxidative stress and ROS initiate and accelerate the progression of atherosclerotic diseases through ED [42]. In cerebrovascular diseases, as intact EC is an important part of normal neurovascular units, the death of ECs will lead to serious damage to the blood-brain barrier and cause various pathophysiological processes, which eventually mediate more extensive programmed cell deaths [43].

EC death such as ferroptosis and pyroptosis [44] have recently been implicated in ED and vascular diseases, although the exact mechanisms remain unclear. The precise role of each mode of cell death needs further elucidation to yield new therapeutic targets that could facilitate EC survival under disease states and explore treatments of various ED-related diseases.

## 4. Ferroptosis of ECs

Ferroptosis occurs in ECs after fine particulate matter (PM2.5) exposure, characterized by intracellular iron overload, lipid peroxidation, GSH depletion, and redox imbalance [45]. Furthermore, iron-bearing nanoparticles, which are abundant in PM, trigger ferroptotic responses by increasing intracellular iron levels [46]. Activation of the p53-xCT (the substrate-specific subunit of system Xc^−^)-GSH axis enhances ferroptosis in human umbilical vein endothelial cells (HUVECs). High glucose (HG) and interleukin-1β (IL-1β) induce ferroptosis with an increase in p53 and a reduction in xCT (SLC7A11) and GSH [47]. GPX4 knockdown in HUVECs increases lipid oxidation and lactate dehydrogenase (LDH) levels while reducing cellular proliferation [48]. Oxidized low-density lipoprotein (Ox-LDL) promotes ED by inducing ferroptosis with decreasing GPX4 and xCT expression [12,49]. Ox-LDL exposure could also induce mitochondrial damage [12,50]. The iron and total ROS content significantly increase, whereas the GSH level decreases [50]. Zinc oxide nanoparticles (ZnONPs) have shown promise in the field of biomedicine [51]. However, ZnONP exposure can cause ferroptosis in vascular ECs. NCOA4-mediated ferritin degradation is required for the ferroptosis of ECs induced by ZnONPs. ROS originating from mitochondria may trigger AMPK-Unc-51-like autophagy activating kinase 1 axis to cause ferritinophagy [52].

**Figure 2:**
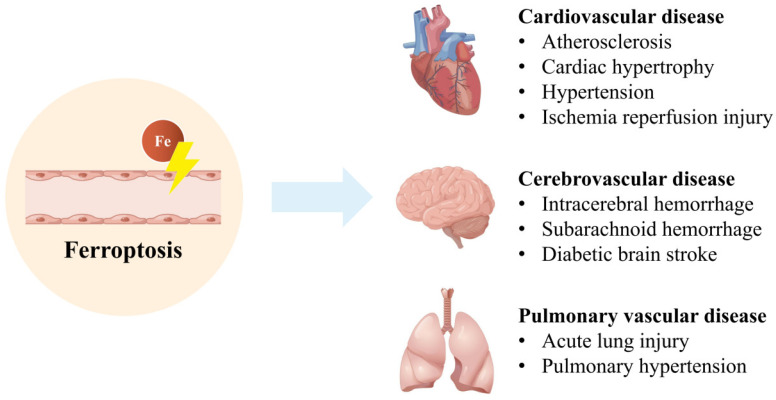
Ferroptosis of ECs is related to multiple vascular diseases, such as AS, stroke, ALI, and so on. Modified from Figdraw (www.figdraw.com, accessed on 22 May 2022).

The ferroptosis of vascular ECs occurs in multiple vascular diseases (Figure 2). The molecular mechanisms of several treatments for inducing or inhibiting ferroptosis of ECs are summarized in Table 2.

### 4.1. Ferroptosis of ECs in Cardiovascular Disease

Many serious vascular diseases have AS as their basic pathological state [69]. AS is mainly caused by lipid deposition, EC damage, vascular smooth muscle cell proliferation, macrophage transformation, and so on [70]. During the onset and progression of AS, ferroptosis may occur. In high-fat diet-induced apolipoprotein E knockout (ApoE^−/−^) mice, ferroptosis inhibitor ferrostatin-1 (Fer-1) alleviated AS lesions, ameliorated lipid peroxidation, reduced iron accumulation, and suppressed cell death and angiogenesis in the thoracic aorta. The effects of inhibition of ferroptosis were also evaluated in ox-LDL-induced mouse aortic EC (MAECs) in vitro. EC injury, lipid peroxidation, cell death, angiogenesis, and ED induced by ox-LDL can all be reduced by Fer-1 [12]. Ox-LDL exposure caused the mitochondrial damage of human coronary artery ECs (HCAECs) and increased iron and total ROS levels, which promoted ferroptosis. Decaprenyl diphosphate synthase subunit 2 (PDSS2) overexpression can suppress the ferroptosis of HCAECs by activating NRF2 pathways [50].

As ED in diabetes leads to the development of cardiovascular complications [71], it is vital to investigate the function and regulatory mechanisms of ferroptosis in diabetes-induced ED. The aorta of db/db mice exhibited increased lipid peroxidation production and iron accumulation. Due to the impairment of xCT expression by p53 activation, there is a reduction in intracellular cystine and GSH production, leading to ferroptosis and ED [47]. A bioinformatic assay identified that ferroptosis and HMOX1 were key factors in atherosclerotic vascular diseases. Additionally, an in vivo study revealed that diabetes greatly contributed to ferroptosis and increased HMOX1 levels. Fer-1 could effectively rescue them. In MAECs and HUVECs, Fer-1 ameliorated HG/high lipid-induced redox imbalance, GSH depletion, and lipid peroxidation. HMOX1 knockdown reduced Fe^2+^ overload, iron content, and ROS and alleviated lipid peroxidation, which attenuated ferroptosis in diabetic human ECs. Genetic/pharmacologic HMOX1 inhibition and other interventions that block ferroptosis, such as Fer-1 administration, may represent potential approaches to treat diabetic AS [55]. Moreover, iron overload may contribute to EC calcification and be related to cell ferroptosis. Cellular calcification can be alleviated with ferroptosis inhibitors [59].

Ferroptosis of ECs also contributes to other cardiovascular diseases, such as cardiac hypertrophy [56,57] and hypertension [58]. The underlying mechanisms of treatments associated with ferroptosis of ECs are shown in Table 2.

### 4.2. Ferroptosis of ECs in Cerebrovascular Disease

The cerebrovascular diseases, intracerebral hemorrhage (ICH) and subarachnoid hemorrhage (SAH), have high morbidity and mortality rates [72,73,74]. The pathological process of hemorrhagic stroke has lately been linked to ferroptosis [75,76,77,78,79]. Additionally, this association was demonstrated in several studies on ECs (Table 2) [61,62,63]. Mouse brain microvascular ECs (BMVECs) bEnd.3 and rat BMVECs are commonly used to study chronic cerebrovascular disease. Gao et al. observed that ferroptosis occurred in the microglia and endothelium after SAH, and this process was facilitated by increasing arachidonate 15-lipoxygenase (ALOX15) levels [63]. Long noncoding RNA (lncRNA) H19 was overexpressed in ICH model cells. H19 knockdown promoted cell proliferation and suppressed ferroptosis of BMVECs by regulating the miR-106b-5p/ACSL4 axis [61]. Individuals with diabetes were not only at higher risk of stroke, but also experienced functional impairment and poor recovery after ischemic brain injury [80,81]. Therefore, several studies focused on ferroptosis of ECs in diabetic cerebrovascular diseases [64,65]. The functional and regulatory roles of maternally expressed gene 3 (Meg3) in diabetic brain ischemic injuries were studied in vitro. By inhibiting GPX4 activity, the Meg3-p53 signaling pathway mediated the ferroptosis of rat BMVECs, induced by oxygen and glucose deprivation (OGD), combined with hyperglycemic reperfusion [64]. In BMVECs derived from male type 2 diabetic rats, deferoxamine (DFO) significantly reduced ferroptosis markers and prevented iron-mediated decline in cell viability [65].

### 4.3. Ferroptosis of ECs in Pulmonary Vascular Disease

ALI is a life-threatening inflammatory disease linked to the dysfunction of the alveolar-capillary barrier caused by various factors [82,83]. Ferroptosis was reported to be crucial in radiation-induced lung injury by mediating the release of inflammatory cytokines [84]. Ferroptosis of lung ECs modulated by piezo-type mechanosensitive ion channel component 1 (PIEZO1)/calcium (Ca^2+^)/calpain signaling was a potential therapeutic mechanism. Vascular endothelial-cadherin (VE-cadherin) knockdown caused ferroptosis-like phenomena in human pulmonary microvascular endothelial cells (HULEC-5a), whereas VE-cadherin overexpression partly decreased ferroptosis [66]. In addition to ALI, ferroptosis of pulmonary artery ECs (PAECs) is also involved in pulmonary hypertension (PH). Pulmonary artery endothelial ferroptosis triggers inflammatory responses through the high-mobility group box 1/Toll-like receptor 4/NACHT, LRR and PYD domains-containing protein 3 (NLRP3) inflammasome signaling pathway. A ferroptosis inhibitor (such as Fer-1) may attenuate PH progression [67].

## 5. Potential Ferroptosis Inhibitors

As ferroptosis of ECs is associated with multiple vascular diseases, ferroptosis inhibition may provide a novel treatment strategy. In addition to lipid peroxidation inhibitors and iron chelators, growing evidence shows that clinical drugs, natural products, and ncRNA intervention may also inhibit ferroptosis of ECs, as summarized in Table 3.

### 5.1. Lipid Peroxidation Inhibitors and Iron Chelators

Lipid peroxidation inhibitors (e.g., Fer-1 and liproxstatin-1) and iron chelators (e.g., DFO and deferiprone (DFP)) can pharmacologically inhibit ferroptosis. Growing evidence shows that Fer-1 rescues lipid peroxidation and ED in several ECs, such as hemangioma ECs, corneal ECs, MAECs, PAECs, and HUVECs [12,45,47,52,67,92,93]. Liproxstatin-1 reverses ferroptosis by scavenging free radicals to prevent PUFA oxidation, whose therapeutic effects were similar to Fer-1 [94,95].

Several studies showed that DFO and DFP could reduce intracellular iron levels and cell ferroptosis [12,45,47,49,52,58,62,65]. DFOs are clinically approved iron chelators that can remove iron by binding with free or protein-bound Fe^3+^ [95] and prevent the onset of lipid peroxidation by inhibiting lipoxygenases (LOXs) [96]. Moreover, they reduced inflammation and the development of atherosclerotic lesions in ApoE-deficient mice, showing cardiovascular protective effects [97]. Iron chelation and possibly ferroptosis inhibition may provide a novel disease-modifying treatment approach in preventing post-stroke cognitive impairment in diabetes and alleviating AS [58,65]. However, DFO is ototoxic and neurotoxic [98]. Therefore, more attention should be paid to compounds with fewer side effects.

### 5.2. Clinical Drugs

In addition to the compounds mentioned above, which are closely related to the crucial mechanism of ferroptosis, some clinical drugs have also shown the potential to inhibit ferroptosis of ECs (Table 3). Fluvastatin may protect ECs from ferroptosis by counteracting the ox-LDL-induced inhibition of GPX4 and xCT expression [49]. Dexmedetomidine and tongxinluo can also protect ECs from ferroptosis [68,85].

### 5.3. Natural Products

Natural products are common resources for drug discovery [99]. Some have been used to manage human diseases [100,101]. Moreover, medical nutritional therapy with healthy foods and their bioactive ingredients has been considered an alternative strategy for the treatment of certain chronic diseases [102]. With the rapid development of studies on ferroptosis, increasing evidence has revealed that natural products can regulate ferroptosis, and are related to cancers, Parkinson’s disease, intracerebral hemorrhage, and other diseases [103,104,105,106]. In terms of ferroptosis in ECs, the potential role of natural products in regulating ferroptosis-mediated diseases requires further study and may be a hot area for future research.

The ethyl acetate-extracted fraction of the total *Ginkgo biloba* flower extract showed an anti-ferroptosis effect in vascular ECs, and luteolin was the active compound in the extract. Luteolin downregulated ACSL4 and upregulated GPX4 in erastin-induced ferroptosis of HUVECs [86]. Astragaloside IV significantly reversed decreased cell activity, elevated iron ion and lipid ROS levels, enhanced cell senescence, and reversed the change in mitochondrial morphology caused by lysophosphatidylcholine treatment. The mechanism may be due to the partial upregulation of SLC7A11 and GPX4 expression levels [87]. A new flavonoid glycoside isolated from *Clematis tangutica*, Apigenin-7-O-β-D-(-6″-p-coumaroyl)-glucopyranoside, attenuated intestinal ischemia-reperfusion injury (IIRI)-induced ROS generation, Fe^2+^ accumulation, and mitochondrial damage, and could inhibit ferroptosis through HMOX1 and monoamine oxidase B (MAO-B) [88]. The active lipophilic ingredient, tanshinone IIA (TSA), which was isolated from the root of *Salvia miltorrhiza* [107], protected cells by modulating intracellular redox status and has been extensively used to treat cardiovascular diseases [108]. He et al. found that TSA protected HCAECs from ferroptosis by activating the NRF2 pathway [60]. After SAH, ferroptosis in the microglia and endothelium was facilitated by treatment with cepharanthine, a type of bisbenzylisoquinoline alkaloid, by downregulating ALOX15 levels [63]. In addition, vitamin E-rich food, such as brown rice, can compensate for the loss of GPX4 by protecting cells from lipid peroxidation [48]. Docosahexaenoic acid (DHA), the primary ingredient of Omega (ω)-3 polyunsaturated fatty acids, can provide numerous benefits for human health [109]. DHA treatment has been identified to upregulate the expression of interferon regulating factor 3 and reduce cellular ferroptosis [57].

Importantly, natural products have the advantages of stable structure, higher safety, low cost, and easy availability, compared with classical ferroptosis inhibitors. Therefore, it is a worthwhile and promising endeavor to explore natural products as ferroptosis inhibitors. Further clinical investigations are urgently required to assess the efficacy and viability of natural products as dietary supplements or prospective medications in vivo because the exactly regulatory targets and molecular mechanisms regulating ferroptosis by natural products remain unclear.

### 5.4. Novel Regulators Associated with Molecular Biology

#### 5.4.1. ncRNAs

lncRNAs are RNAs >200 nt in length, which act as regulators of almost every cellular process, physiological condition, and various human diseases [110]. Some lncRNAs act as miRNA sponges and negatively regulate miRNAs. miRNAs with a size of ~20 nt can negatively regulate gene expression by binding a target mRNA and inhibiting its translation or promoting its degradation [111,112].

Links between ncRNAs and endothelial ferroptosis have opened up a new realm of therapeutic opportunities. lncRNA H19 knockdown promoted cell viability and inhibited ferroptosis of BMVECs by regulating the miR-106b-5p/ACSL4 axis, which may be a potential therapeutic target for ICH [61]. By modulating GPX4 transcription and expression, lncRNA Meg3 mediated ferroptosis induced by OGD combined with hyperglycemia in rat BMVECs via the p53/GPX4 axis. Therefore, Meg3 may be an attractive target for treating diabetic brain ischemia [64]. Targeting the lncRNA Meg8/miR-497-5p/Notch receptor 2 signaling pathway may inhibit the ferroptosis of hemangioma ECs [93].

For miRNAs, miR-132 promotes AS by inducing mitochondrial oxidative stress-mediated ferroptosis and downregulating GPX4 [53]. miR-17-92 targeting the zinc lipoprotein A20-ACSL4 axis protects HUVEC from erastin-induced ferroptosis [89].

#### 5.4.2. Exosomes (Exos) or Extracellular Vesicles (EVs)

Endothelial progenitor cells (EPCs) are involved in maintaining endothelial homeostasis and forming new vessels [113]. EVs are small membrane-bound vesicles secreted by almost all cell types and mediate intercellular communication by transferring bioactive molecules, such as miRNAs [114]. Exos are EVs that deliver miRNAs, mRNAs, and proteins, which are important for communication from organ to organ and cell to cell [115]. EPC-derived Exos (EPC-Exos) had a protective effect on ECs in a mouse model of diabetes [116]. In irradiated fibroblasts, one study identified the regulation of Exos in cell ferroptosis [117]. Moreover, miRNA itself can also relieve ferroptosis [118]. Therefore, the role of EPC-EVs or EPC-Exos carrying a miRNA in ferroptosis of ECs deserves further investigation and may confer novel insights for clinical management.

EPC-EVs transferred miR-199a-3p to inhibit specificity protein 1 (SP1), further repress ferroptosis of ECs, and alleviate AS occurrence [54]. EPC-derived Exos transferred miR-30e-5p, which inhibited SP1 and activated the AMPK pathway to regulate erastin-induced ferroptosis in HUVECs [90]. As a widely expressed transcription factor that regulates the inflammatory repair process, SP1 is a key target for treating atherosclerotic vascular diseases [119,120].

#### 5.4.3. Other Targets

NRF2, a transcription factor, regulates several metabolic mechanisms, including proteostasis, xenobiotic/drug metabolism, iron/heme metabolism, carbohydrate and lipid metabolism, apoptosis, and ferroptosis, as well as the cellular antioxidant response [121]. Two of the most important targets inhibiting ferroptosis, xCT, and GPX4, are established downstream transcriptional targets of NRF2. Therefore, NRF2 is essential for reducing lipid peroxidation and ferroptosis [122]. By promoting the activation of NRF2 pathways, PDSS2 overexpression prevents HCAECs from ferroptosis. Thus, PDSS2 may be protective in AS [50].

In addition, HMOX1 upregulation promotes ferroptosis in diabetic AS. HMOX1 inhibition via genetic or pharmacological means may have the potential to attenuate ferroptosis [55]. PIEZO1/Ca^2+^/calpain signaling modulates ferroptosis of lung ECs, which is a potential therapeutic mechanism for treating radiation-induced lung injury [66]. Tripartite motif-containing 46 and GPX4 form a regulatory pathway that controls HG-induced ferroptosis of human retinal capillary ECs. Thus, inhibiting this pathway or maintaining GPX4 expression enables cells to resist damage caused by HG [91]. Moreover, elabela, a second endogenous ligand for the apelin receptor, can antagonize angiotensin II-mediated cardiac microvascular ECs (CMVECs) ferroptosis by modulating the IL-6/signal transducer and activator of transcription 3/GPX4 pathway [58].

### 5.5. Nanomaterials

Nanoparticles have already been used as drug delivery systems and have greatly extended the range of drug carriers. For enhancing in vivo siRNA delivery, nanotechnology has shown great potential [98].

lncRNA AAB sponged and sequestered miR-30b-5p to induce the imbalance of matrix metalloproteinase 9/TIMP metallopeptidase inhibitor 1, which enhanced TFR1 activation and eventually caused the ferroptosis of CMVECs. Shi et al. developed a delivery system that effectively transported silenced lncRNA AAB (si-AAB) using the neutrophil membrane (NM)-camouflaged mesoporous silica nanocomplex (MSN). NM+si-AAB+MSN inhibited ferroptosis of CMVECs by miR-30b-5p upregulation. An in vivo study showed that it could repair the cardiac microvascular endothelial injury in cardiac hypertrophy. The MSN core has a high siRNA loading capacity and effectively protects the nanocomplex from degradation in body fluids. At the endothelial injury site, the si-AAB is released long-term to prevent ferroptosis of CMVECs, which improves cardiac microvascular function [56].

## 6. Conclusions

Ferroptosis occurs in ECs, according to a growing body of studies. In this review, ferroptosis of ECs, their role in vascular diseases, and potential regulators were summarized. Ferroptosis of ECs has an intimate connection with cardiovascular, cerebrovascular, and pulmonary vascular diseases. Lipid peroxidation inhibitors, iron chelators, and some clinical drugs have shown their potential as inhibitors of ferroptosis in ECs. Natural products, ncRNAs, and other novel targets involved in the ferroptosis pathway also conferred new insights for clinical management. Exos or EVs and nanomaterials that deliver key regulators require further investigation. More studies on regulatory ferroptosis of ECs will broaden the pathogenesis of vascular diseases and provide novel treatment strategies for multiple diseases.

## Figures and Tables

**Table 1:** The main features of various RCD subtypes.

Type	Morphological Features	Biochemical Features	Immune Features
Necroptosis	Cell swelling; plasma membrane rupture; moderate chromatin condensation	RIPK1, RIPK3, and MLKL activation; cytosolic necrosome formation	Mostly pro-inflammatory
Apoptosis	Cell rounding-up; pseudopode retraction; cellular and nuclear volume reduction; plasma membrane blebbing; nuclear fragmentation; apoptotic body formation	Caspase activation; DNA fragmentation; ΔΨm dissipation; phosphatidylserine exposure	Often anti-inflammatory and immune silent; sometimes elicits an immune response
Autophagy	Double-membraned autophagic vacuole formation	Increased lysosomal activity	Mostly anti-inflammatory
Pyroptosis	Cell swelling; pore formation; membrane rupture; massive leakage of cytoplasmic components	Caspase-1/4/5/11 activation and proinflammatory cytokine release	Pro-inflammatory
Ferroptosis	Smaller mitochondria; mitochondria crista reduction; mitochondrial membrane density elevation; increased mitochondrial membrane rupture	Iron accumulation; lipid peroxidation	Pro-inflammatory

RIPK: receptor-interacting serine/threonine kinase; MLKL: mixed lineage kinase domain-like pseudokinase; ΔΨm: mitochondrial membrane potential.

**Table 2:** Treatment associated with ferroptosis of ECs in vascular diseases.

Category	Vascular Disease	Cells	Treatment Leading to Ferroptosis	Treatment Inhibiting Ferroptosis and Its Mechanism	Ref.
Cardiovascular disease	Atherosclerosis	HCAEC	Ox-LDL	Overexpression of PDSS2: ↑ NRF2 pathway	[50]
	Atherosclerosis	HUVEC	MiR-132	/	[53]
	Atherosclerosis	MAEC	Ox-LDL	Fer-1: ↑ SCL7A11 and GPX4 DFO: ↓ iron	[12]
	Atherosclerosis	MAEC	Ox-LDL	EPC-EVs carrying miR-199a-3p targeting SP1	[54]
	Diabetic atherosclerosis	HUVEC; MAEC obtained from ApoE^−/−^ mice	High glucose/high lipids	Fer-1; knockdown of HMOX1	[55]
	Cardiac hypertrophy	CMVEC	Ang II	Neutrophil-like cell membrane-coated siRNA of lncRNA AABR07017145.1: ↑ miR-30b-5p targeting TIMP1, ↓ TFR-1	[56]
	Cardiac hypertrophy	CMVEC	Ang II	DHA: ↑ IRF3, ↑ SLC7A11/ALOX12	[57]
	Hypertension	CMVEC	Ang II	Elabela: ↓ IL-6/STAT3 pathway, ↑ xCT/GPX4 pathway	[58]
	Endothelial cell calcification	HUVEC	FeSO_4_	DFO: ↓ iron; ferrostatin	[59]
	/	HCAEC	/	Tanshinone IIA: ↑ NRF2 pathway	[60]
Cerebrovascular disease	ICH	RBMVEC	LncRNA H19	Knockdown of lncRNA H19: ↑ miR-106b-5p, ↓ ACSL4	[61]
	ICH	bEnd.3	Iron	DFO-HCC-PEG: combined iron chelator therapy	[62]
	Early brain injury after SAH	bEnd.3	RSL3	Cepharanthine: ↓ ALOX15	[63]
	Diabetic brain stroke	RBMVEC	Oxygen and glucose deprivation + hyperglycemic reperfusion	Knockdown of lncRNA Meg3: ↓ p53, ↑ GPX4	[64]
	Post-stroke cognitive impairment in diabetes	RBMVEC	Iron(III) sulfate	Deferoxamine: iron chelator therapy	[65]
Pulmonary vascular disease	Lung injury: radiation-induced lung injury	HULEC-5a	Ionizing radiation: ↑ PIEZO1, ↑ intracellular calcium concentration and calpain activity, ↓ VE-cadherin	GsMTx4, a specific inhibitor of PIEZO1; PD151746, a selective calpain inhibitor; overexpression of VE-cadherin	[66]
	Pulmonary hypertension	PAEC	PAEC from monocrotaline-induced pulmonary hypertension rats	Fer-1: ↓ HMGB1 and TLR4, ↓ NLRP3 inflammasome and proinflammatory cytokines	[67]
	COPD complicated with atherosclerosis	HPMEC	Cigarette smoke extract; homocysteine	Tongxinluo: ↑ GPX4 and FSP1, ↓ ACSL4	[68]

↑ indicates elevation or activation; ↓indicates reduction or suppression. ICH: intracerebral hemorrhage; SAH: subarachnoid hemorrhage; LPS: lipopolysaccharide; COPD: chronic obstructive pulmonary disease; HCAEC: human coronary artery endothelial cell; MAEC: mouse aortic endothelial cell; CMVEC: cardiac microvascular endothelial cell; RBMVEC: rat brain microvascular endothelial cell; bEnd.3: mouse brain microvascular endothelial cell; HULEC-5a: human pulmonary microvascular endothelial cell; PAEC: pulmonary artery endothelial cell; HPMEC: human pulmonary microvascular endothelial cell; Ang II: angiotensin II; LncRNA: long non-coding RNA; PIEZO1: piezo type mechanosensitive ion channel component 1; VE-cadherin: vascular endothelial-cadherin; PDSS2: prenyldiphosphate synthase subunit 2; Fer-1: ferrostatin-1; DFO: deferoxamine; EPC-EVs: endothelial progenitor cells-secreted extracellular vesicles; siRNA: small interfering RNA; SP1: specificity protein 1; TIMP1: TIMP metallopeptidase inhibitor 1; DHA: docosahexaenoic acid; IRF3: interferon regulating factor 3; ALOX12: arachidonate 12-lipoxygenase, 12S Type; IL-6: interleukin-6; STAT3: signal transducer and activator of transcription 3; PEG-HCC: poly(ethylene glycol)-conjugated hydrophilic carbon clusters; ALOX15: arachidonate 15-lipoxygenase; Meg3: maternally expressed gene 3; HMGB1: high-mobility group box 1; TLR4: toll-like receptor 4; NLRP3: NACHT, LRR and PYD domains-containing protein 3.

**Table 3:** Potential inhibitors of ferroptosis in ECs.

Potential Inhibitors	Cells	Inhibitors	Mechanisms	Ref.
Clinical drugs	HUVEC	Fluvastatin	↑ GPX4, ↑ xCT	[49]
	RPVEC	Dexmedetomidine	↑ NRF2, ↓ mitochondrial fission	[85]
	HPMEC	Tongxinluo	↑ GPX4 and FSP1, ↓ ACSL4	[68]
Natural products	HUVEC	α-tocopherol (vitamin E) and extract of brown rice	↑ GPX4	[48]
	HUVEC	Luteolin	↓ ACSL4, ↑ GPX4	[86]
	HUVEC	Astragaloside IV	Partially ↑ SLC7A11 and ↑ GPX4	[87]
	HUVEC	Apigenin-7-O-β-D-(-6”-p-coumaroyl)-glucopyranoside	↓ HMOX1, ↓ MAO-B	[88]
	HCAEC	Tanshinone IIA	↑ NRF2	[60]
	CMVEC	DHA	↑ IRF3	[57]
	bEnd.3	Cepharanthine	↓ ALOX15	[63]
Nanomaterials	CMVEC	Neutrophil membrane + si-AAB + mesoponanocomplex	↑ miR-30b-5p, ↓ TIMP1, ↓ TFR-1	[56]
ncRNAs	BMVEC	LncRNA H19 knockdown	↑ miR-106b-5p,↓ ACSL4	[61]
	BMVEC	LncRNA Meg3-siRNA	↓ Meg3, ↓ p53, ↑ GPX4	[64]
	HUVEC	Overexpression of miRNA-17-92	↓ A20, ↓ ACSL4	[89]
EVs or Exos	MAEC	Mouse EPC-EVs	↑ miR199a-3p, ↓ SP1	[54]
	HUVEC	Human umbilical vein blood EPC-Exos	↑ miR-30e-5p, ↓ SP1, ↑ AMPK	[90]
Other novel targets	HCAEC	Overexpression of PDSS2	↑ NRF2	[50]
	HUVEC	Knockdown of HMOX1	↓ iron, ↓ ROS, ↓ lipid peroxidation, ↑SLC7A11, ↑GPX4	[55]
	HULEC-5a	GsMTx4, a specific inhibitor of PIEZO1; PD151746, a selective calpain inhibitor; Overexpression of VE-cadherin	↓ PIEZO1/Ca^2+^/calpain, ↑ VE-Cadherin	[66]
	HRCEC	Knockdown of TRIM46	↑ GPX4	[91]
	CMVEC	Elabela	↓ IL-6/STAT3 pathway, ↑ xCT/GPX4 pathway	[58]

↑ indicates elevation or activation; ↓indicates reduction or suppression. RPVEC: rat pulmonary vein endothelial cell; HRCEC: human retinal capillary endothelial cell; AAB: long non-coding RNA AABR07017145.1; Exo: exosome; TRIM46: tripartite motif containing 46; MAO-B: monoamine oxidase B; A20: zinc lipoprotein A20; SP1: specificity protein 1.

## Data Availability

Not applicable.

## Abbreviations

ΔΨm: mitochondrial membrane potential; 4-HNE: 4-hydroxy-2-nonenal; A20: zinc lipoprotein A20; AAB: long non-coding RNA AABR07017145.1; ACC: acetyl coenzyme A carboxylase; ACSL4: acyl-CoA synthetase long-chain family member 4; ALI: acute lung injury; ALOX12: arachidonate 12-lipoxygenase, 12S Type; ALOX15: arachidonate 15-lipoxygenase; AMPK: adenosine monophosphate-activated protein kinase; Ang II: angiotensin II; ApoE: apolipoprotein E; AS: atherosclerosis; bEnd.3: mouse brain microvascular endothelial cell; BH2: dihydrobiopterin; BH4: tetrahydrobiopterin; BMVEC: brain microvascular EC; Ca^2+^: calcium; CMVEC: cardiac microvascular endothelial cell; CoA: coenzyme A; COPD: chronic obstructive pulmonary disease; CoQ_10_: coenzyme Q_10_; CoQ_10_H2: the reduced form of coenzyme Q_10_, ubiquinol; DFO: deferoxamine; DFP: deferiprone; DHA: docosahexaenoic acid; DHFR: dihydrofolate reductase; EC: Endothelial cell; EPC: endothelial progenitor cell; EPC-Exo: EPC-derived exosome; EPC-EV: EPC-secreted extracellular vesicle; EV: extracellular vesicle; Exo: exosome; Fe^2+^: ferrous ion; Fer-1: ferrostatin-1; FSP1: ferroptosis suppressor protein 1; GCH1: guanosine 5’-triphosphate cyclohydrolase 1; GPX4: glutathione peroxidase 4; GSH: glutathione; GSSG: glutathione disulfide; HCAEC: human coronary artery endothelial cell; HMGB1: high-mobility group box 1; HMOX1: heme oxygenase 1; HPMEC: human pulmonary microvascular endothelial cell; HRCEC: human retinal capillary endothelial cell; HULEC-5a: human pulmonary microvascular endothelial cell; HUVEC: human umbilical vein endothelial cell; ICH: intracerebral hemorrhage; IIRI: intestinal ischemia-reperfusion injury; IL-6: interleukin-6; IRF3: interferon regulating factor 3; Keap 1: Kelch-like ECH-associated protein 1; L: lipid; LDH: lactate dehydrogenase; lncRNA: long noncoding RNA; LOX: lipoxygenase; LPCAT3: lysophosphatidylcholine acyltransferase 3; LPS: lipopolysaccharide; MAEC: mouse aortic endothelial cell; MAO-B: monoamine oxidase B; MDAs: malondialdehydes; Meg3: maternally expressed gene 3; MLKL: mixed lineage kinase domain-like pseudokinase; MSN: mesoporous silica nanocomplex; NADH: nicotinamide adenine dinucleotide; NADPH: nicotinamide adenine dinucleotide phosphate; NCOA4: nuclear receptor coactivator 4; NLRP3: NACHT, LRR and PYD domains-containing protein 3; NM: neutrophil membrane; NRF2: nuclear factor erythroid 2-related factor 2; -OHs: alcohols; -OOHs: hydroperoxides; Ox-LDL: oxidized low-density lipoprotein; OXPHOX: oxidative phosphorylation; PAEC: pulmonary artery endothelial cell; PDSS2: prenyldiphosphate synthase subunit 2; PEG-HCC: poly(ethylene glycol)-conjugated hydrophilic carbon clusters; PH: pulmonary hypertension; PIEZO1: piezo type mechanosensitive ion channel component 1; PL: phospholipid; PM2.5: particulate matter <2.5 mum in diameter; POR: cytochrome P450 reductase; PUFA: polyunsaturated fatty acid; RBMVEC: rat brain microvascular endothelial cell; RCD: regulated cell death; RIPK: receptor-interacting serine/threonine kinase; ROS: reactive oxygen species; RPVEC: rat pulmonary vein endothelial cell; RSL3: Ras-selective lethal small molecule 3; SAH: subarachnoid hemorrhage; si-AAB: silenced lncRNA AAB; siRNA: small interfering RNA; SLC3A2: solute carrier family 3 member 2; SLC7A11: solute carrier family 7 member 11; SP1: specificity protein 1; STAT3: signal transducer and activator of transcription 3; STEAP3: six-transmembrane epithelial antigen of prostate 3; TAC: tricarboxylic acid; TF: transferrin; TFR1: transferrin receptor 1; TIMP1: TIMP metallopeptidase inhibitor 1; TLR4: toll-like receptor 4; TRIM46: tripartite motif containing 46; TSA: tanshinone IIA; VE-cadherin: vascular endothelial-cadherin; ZnONPs: zinc oxide nanoparticles.
